# The Cropping Obstacle of Garlic Was Associated With Changes in Soil Physicochemical Properties, Enzymatic Activities and Bacterial and Fungal Communities

**DOI:** 10.3389/fmicb.2022.828196

**Published:** 2022-03-30

**Authors:** Jinyang Yu, Yihao Liu, Zuyu Wang, Xiaohui Huang, Dan Chai, Yunfu Gu, Ke Zhao, Xiumei Yu, Zhengbin Shuai, Hanjun Liu, Xiaoping Zhang, Petri Penttinen, Qiang Chen

**Affiliations:** ^1^College of Resources, Sichuan Agricultural University, Chengdu, China; ^2^Institute of Horticulture, Chengdu Academy of Agriculture and Forestry Sciences, Chengdu, China; ^3^Safety and Environmental Protection Quality Supervision and Testing Research Institute, CNPC Chuanqing Drilling Engineering Co., Ltd., Guanghan, China

**Keywords:** garlic, cropping obstacles, soily acidification, soil enzyme activity, soil microbial community

## Abstract

**Aims:**

In garlic cultivation, long-time monoculture has resulted in continuous-cropping obstacles. However, the cause has not been studied to date.

**Methods:**

We analyzed soils from garlic fields in Pengzhou, China, to determine continuous-cropping obstacle related changes in soil physicochemical properties and enzyme activities, and in the diversity and composition of bacterial and fungal communities. Furthermore, we examined the relationships between soil properties and the bacterial and fungal communities.

**Results:**

The soil pH and the soil catalase, urease, invertase, and polyphenol oxidase activities were lower in the cropping obstacle soil than in the healthy control soil. The richness and diversity of the bacteria were lower in the cropping obstacle soil than in the control. The bacterial and fungal communities in the cropping obstacle soil were clearly different from those in the control soil. The differences in bacterial communities between the cropping obstacle soil and the control soil were associated with differences in pH and available potassium content. The taxa with higher relative abundances in the cropping obstacle soils included potential plant pathogens and the taxa with lower relative abundances included potential plant growth promoters.

**Conclusion:**

The enrichment of plant pathogens and the depletion of plant growth promoting fungi may have contributed to the poor growth of garlic in the cropping obstacle soil. The enzyme activity and microbial community differences were associated with acidification that was likely an important factor in the deterioration of the soil ecological environment and the garlic cropping obstacle. The results provide information to guide agricultural practices in cultivating garlic.

## Highlights

- Soil acidification was the primary factor correlating with garlic cropping obstacle.
- The activities of catalase, urease, invertase and polyphenol oxidase were lower in the cropping obstacle soil than in the healthy control soil.
- The enrichment of plant pathogens and the depletion of plant growth promoting fungi may have contributed to the garlic cropping obstacle.
- The assembly of bacterial communities was dominated by stochastic processes in garlic cropping obstacle soil.

## Introduction

Garlic (*Allium sativum* L.) is rich in nutrients and has an appealing flavor and antibacterial properties; thus, it has been used as a seasoning, functional food, and traditional medicine for thousands of years worldwide (Corzo-Martinez et al., 2007; Liu et al., 2020). Currently, Asia, Europe, and Latin America are the main garlic production regions. In China, the garlic cropping area has reached 830 km^2^ and the annual yield is approximately 2.3 million tons (FAO, 2019). Growing demand for yields and the limited arable land have resulted in garlic production with high cropping intensity and monocultures over long periods. Generally, long-term monoculture continuous cropping decreased crop yield and quality a phenomenon known as cropping obstacle (Zhu et al., 2018). The cropping obstacle may include numerous biotic and abiotic factors, e.g., changes in microbial communities (Dong et al., 2017), enriched soil-borne plant pathogens (Liu et al., 2014), declines in soil enzyme activity (Fu et al., 2017), changes in soil physicochemical properties (Kaur and Singh, 2014; Perez-Brandan et al., 2014; Li et al., 2016), and autotoxicity of plants (van Wyk et al., 2017).

Soil microorganisms play an important role in agricultural production by maintaining soil quality and affecting nutrient cycling (Blagodatskaya and Kuzyakov, 2013; Sun et al., 2015; Beckers et al., 2017). In some instances, the interactions between soil microbial communities and soil properties modulate plant health through affecting pathogens (Wei et al., 2015). Cropping obstacles have been associated with soil bacterial and fungal communities (Gao et al., 2019, 2021; Xi et al., 2019). For example, decreases in diversity and abundance of beneficial soil microbes and an increase in pathogenic fungi such as *Fusarium* and *Verticillium* were associated with sugarcane cropping obstacle (Pang et al., 2021). However, the relationships between microbial communities and continuous cropping obstacles are not necessarily similar across diverse crops and environmental conditions, thus more studies are called for.

Soil enzymes, i.e., the extracellular enzymes produced by the microorganisms, are considered as indicators of soil fertility (Zeng et al., 2007; Paz-Ferreiro and Fu, 2016). The physicochemical properties of soil affect the composition of soil microbial communities and the enzyme activities (Liu et al., 2021). Among the soil physicochemical properties, soil pH is considered as a master variable in affecting the microbial communities (Fierer and Jackson, 2006). Evidently, the changes brought on by continuous cropping, e.g., the often detected acidification, have resulted in changes in the soil microbial communities and soil enzymatic activities (Gao et al., 2019, 2021; Zheng et al., 2019).

Pengzhou in the Chengdu Plain is one of the five major vegetable cultivation bases in China, with a garlic cultivation history of more than 30 years. In Pengzhou, garlic is commonly grown in rotation with spring rice. In recent years, the long-time monoculture has resulted in continuous-cropping obstacles. The symptoms of the obstacle include yellowing of the leaves starting from the tips and edges, stagnated growth of roots and plant, at its worst, dying of the plants. The continuous-cropping obstacles appear commonly on patches in a field and sometimes on an entire field.

To our knowledge, the continuous-cropping obstacles in garlic cultivation have not been studied to date. We analyzed soils from garlic fields in Pengzhou, with the aim to determine continuous-cropping obstacle related changes in (1) soil physicochemical properties and enzyme activities, and in (2) the diversity and composition of bacterial and fungal communities. Furthermore, we examined (3) the relationships between soil properties and the bacterial and fungal communities. We hypothesized that the continuous cropping obstacle would be associated with soil acidification that would further affect the soil microorganisms and decrease enzyme activities. The results provide information to guide agricultural practices in cultivating garlic.

## Materials and Methods

### Site Description and Field Sampling

The field experiment was performed in a garlic production area in Pengzhou (31°02′31″N-31°05′39″N, 103°50′51″E-103°59′42″E), Chengdu Plain, China, that is in the northern part of the subtropical humid climate zone, with a mean annual temperature of 15.7°C, precipitation of 960 mm and approximately 1,180 h of sunshine. The soil on the site is fertile paddy soil. Garlic was grown in rice-garlic rotation and covered with straw after sowing.

**FIGURE 1:**
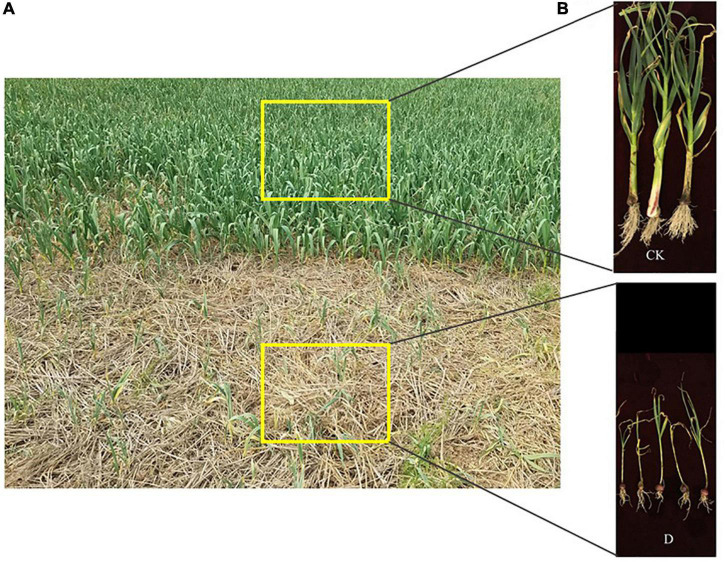
**(A)** A garlic field with cropping obstacle (foreground) and healthy garlic plants (background). **(B)** Garlic plants grown in the healthy control soil (CK) and in the cropping obstacle soil (D).

Samples were collected in March 2018 from seven fields with an area of 700–1,000 m^2^. In each field, three 60–100 m^2^ plots were randomly selected in areas with healthy garlic plants (CK) and in areas with cropping obstacles (D) (Figure 1). Five topsoil (0–20 cm) subsamples from a 10 cm circle around the plants were combined into a composite sample, an appropriate amount of homogenized soil was retained by quartering, and placed into sterile plastic bags that were sealed. The bags were kept on ice and transported to the laboratory immediately after sampling. Roots and other debris were removed from the soil samples and the samples were divided into two parts for immediate DNA extraction and physicochemical analyses.

### Garlic Growth and Root Activity Analyses

The growth of the plants was assessed by measuring shoot and root lengths and the fresh weight of aboveground and root biomasses. Root activity was determined using 2,3,5-triphenyl tetrazolium chloride (TTC) staining (Comas et al., 2008).

### Soil Physicochemical and Enzyme Activity Analyses

Prior the analyses, the soil samples were air-dried naturally in a cool and ventilated place. Soil pH was measured with a pH meter in a 1:2.5 soil to water slurry. Soil organic carbon content (SOC) was determined using the K_2_Cr_2_O_7_ oxidization method. Soil total (TN) and available nitrogen (AN), available phosphorus (AP) and available potassium (AK) contents were determined using Kjeldahl digestion, alkaline hydrolysis diffusion method and molybdenum blue method and flame photometry, respectively.

Soil enzyme activity analyses were performed as described earlier (Guan et al., 1986). Briefly, urease was assayed by colorimetric analysis of sodium phenate-sodium hypochlorite, acid phosphatase was colorimetrically estimated using disodium phenyl phosphate, invertase was colorimetrically determined by DNS based on the decreasing sugar content, phenol oxidase was determined by the pyrogallol colorimetric method, and catalase activity was assayed by potassium permanganate titration.

### DNA Extraction, Amplification, and Sequencing

Total genomic DNA was extracted from 0.60 to 0.90 g fresh weight soils (corresponding to 0.50 g dry weight) using Fast DNA^®^ SPIN for Soil Kit (MP BIO Laboratories, California, United States) according to the manufacturer’s instructions. The quantity and quality of extracted DNA were estimated using a NanoDrop NC2000 spectrophotometer (Thermo Fisher Scientific, Waltham, MA, United States) and agarose gel electrophoresis, respectively. DNA samples were stored at −20°C.

The V3–V4 region of bacterial 16S rRNA gene was amplified using the primers 338F (5′-ACTCCTACGGGAGGCAGCA-3′) and 806R (5′-GGACTACHVGGGTWTCTAAT-3′). The fungal ITS1 region was amplified using the primers ITS5F (5′-GGAAGTAAAAGTCGTAACAAGG-3′) and ITS1R (5′-GCTGCGTTCTTCATCGATGC-3′) using the Q5 High-Fidelity DNA Polymerase (New England Biolabs, Ipswich, MA). Sample-specific 7-bp barcodes were incorporated into the primers for multiplex sequencing. The reaction mixture of total volume 25 μl comprised the following: 5 μl of 5 × reaction buffer, 5 μl of 5 × GC buffer, 2 μl of 2.5 μM dNTPs, 1 μl of forward primer (10 μM), 1 μl of reverse primer (10 μM), 2 μl of DNA Template, 0.25 μl of Q5 DNA polymerase, and 8.75 μl of ddH_2_O. The polymerase chain reaction (PCR) was performed under the following cycling conditions: initial denaturation at 98°C (2 min), denaturation at 98°C (15 s), annealing at 55°C (30 s), extension at 72°C (30 s), and a final extension of 72°C for 5 min, for 25–30 cycles. PCR amplicons were purified with Vazyme VAHTSTM DNA Clean Beads (Vazyme, Nanjing, China) and quantified using the Quant-iT PicoGreen dsDNA Assay Kit (Invitrogen, Carlsbad, CA, United States). Amplicons were pooled in equal amounts, and paired-end 2 × 250 bp sequencing was performed using the Illumina MiSeq platform with MiSeq Reagent Kit v3 at Shanghai Personal Biotechnology Co., Ltd. (Shanghai, China).

### Sequence Analysis

Sequences were processed with QIIME 2 (Bolyen et al., 2019) according to the official tutorial^1^ with slight modifications. Briefly, raw sequence data were demultiplexed using the demux plugin followed by removing the primers with cut adapt plugin (Martin, 2011). Quality filtering, denoising, merging and chimera removal were performed using the DADA2 plugin (Callahan et al., 2016). Non-singleton amplicon sequence variants (ASVs) were aligned with mafft (Katoh et al., 2002) and used to construct a phylogeny with fasttree2 (Price et al., 2009). Taxonomy was assigned to ASVs using the classify-sklearn naïve Bayes taxonomy classifier in feature-classifier plugin (Bokulich et al., 2013) against the SILVA Release 132 and UNITE Release 8.0 databases (Koljalg et al., 2013). Taxonomic compositions were visualized using MEGAN and GraPhlAn (Asnicar et al., 2015). Chao1 and Shannon alpha diversity indices were calculated using the ASV table in QIIME2.

### Statistical Analyses

Statistical analyses were performed using QIIME2, SPSS 21 (Version 21.0, SPSS Inc., Chicago, IL, United States) and R v.3.6.1.^2^ Differences in plant and soil properties were tested using Student’s *t*-test. The associations between garlic growth parameters and soil properties were analyzed using Pearson correlation. Differences in alpha diversity indices between treatments were tested using Kruskal-Wallis test and visualized as box plots. Beta diversity was analyzed based on Bray-Curtis dissimilarity and visualized using non-metric multidimensional scaling (NMDS) (Ramette, 2007). The differences in community compositions were tested using permutational multivariate analysis of variance (PERMANOVA) (McArdle and Anderson, 2001). Differential abundance of taxa was tested using linear discriminant analysis effect size (LEfSe) analysis (Segata et al., 2011). The relationships between environmental factors and microbial community structure were analyzed using distance-based redundancy analysis (dbRDA) in the “vegan” package in R v2.5.6 (Oksanen et al., 2020).

The weighted β nearest taxon index (βNTI) and Bray-Curtis-based Raup-Crick (RC_bray_) values were calculated via a null model methodology to differentiate the ecological processes that regulate microbial community assembly (Stegen et al., 2013, 2015). The βNTI was quantified by determination of the standard deviation between an observed level and the null distribution of the mean nearest taxon distance metric (βMNTD). The βMNTD and RC_bray_ were calculated using the R packages “picante” and “vegan,” respectively (Kembel et al., 2010). Specifically, β-NTI > 2 indicated variable selection, and β-NTI < −2 indicated homogeneous selection; at |β-NTI| < 2, deterministic processes were associated with dispersal limitation when RC_bray_ > 0.95, homogeneous dispersal when RC_bray_ < −0.95, and undominated when |RC_bray_| < 0.95 (Stegen et al., 2015; Jiao et al., 2020; Luan et al., 2020).

## Results

### The Properties of Garlic Plants and Soils

The biomasses, root and shoot lengths and root activities of cropping obstacle plants were lower than those of healthy plants, in which root activities decreased by 61.16% (*P* < 0.05) (Table 1). The pH was lower and AK content higher in the cropping obstacle soil than in the control (*P* < 0.05) (Table 2). Thus, the soil pH correlated positively and AK content negatively with the garlic growth parameters (*P* < 0.01) (Supplementary Table 1). The activities of soil catalase, urease, polyphenol oxidase and invertase were lower and the activity of acid phosphatase was higher in the cropping obstacle soil than in the control (*P* < 0.05) (Table 3).

**TABLE 1:** The properties of garlic plants grown in cropping obstacle soil (D) and healthy control soil (CK).

Item tested	D	CK
Shoot length (cm⋅plant^–1^)	26.34 ± 4.21	71.69 ± 5.14*
Maximum root length (cm⋅plant^–1^)	4.85 ± 1.03	13.87 ± 1.91*
Aboveground biomass (g⋅plant^–1^⋅FW)	4.65 ± 0.86	40.73 ± 11.04*
Root biomass (g⋅plant^–1^⋅FW)	0.34 ± 0.12	4.11 ± 1.56*
Root activity (μg⋅g^–1^ ⋅h^–1^⋅FW)	23.54 ± 5.84	60.61 ± 9.83*

Values are means ± standard deviation. *In a row indicate statistically significant difference at P < 0.05 (Student’s t-test).

**TABLE 2:** The properties of cropping obstacle soil (D) and healthy control soil (CK).

	D	CK
pH	5.09 ± 0.19	6.02 ± 0.46*
WC (%)	28.31 ± 1.99	27.02 ± 4.38
AP (mg/kg)	58.81 ± 5.87	55.58 ± 11.13
AK (mg/kg)	250.09 ± 26.49*	194.14 ± 28.35
AN (mg/kg)	65.40 ± 6.26	65.25 ± 8.24
TN (g/kg)	1.86 ± 0.16	1.86 ± 0.25
SOC (g/kg)	34.97 ± 6.61	38.38 ± 9.09

Values are means ± standard deviation. *In a row indicate statistically significant difference at P < 0.05 (Student’s t-test).

*WC, soil water content; AP, available phosphorus content; AK, available potassium content; AN, available nitrogen content; TN, total nitrogen content; SOC, soil organic carbon content.*

**TABLE 3:** Soil enzyme activities in cropping obstacle soil (D) and healthy control soil (CK).

Enzyme	D	CK
Catalase (KMnO_4_ ml/g⋅20 min)	0.54 ± 0.09	0.92 ± 0.23*
Urease (NH_3_-N mg/g⋅24 h)	0.21 ± 0.02	0.31 ± 0.06*
Polyphenol oxidase (Purple gallate mg/g⋅2 h)	0.88 ± 0.18	1.99 ± 0.49*
Acid phosphatase (Phenol mg/g⋅24 h)	1.98 ± 0.38*	1.64 ± 0.21
Invertase (Glucose mg/g⋅24 h)	2.52 ± 0.69	3.57 ± 0.52*

Values are means ± standard deviation (n = 21). *In a row indicate statistically significant difference at P < 0.05 (Student’s t-test).

### Changes in Bacterial and Fungal Communities

**FIGURE 2:**
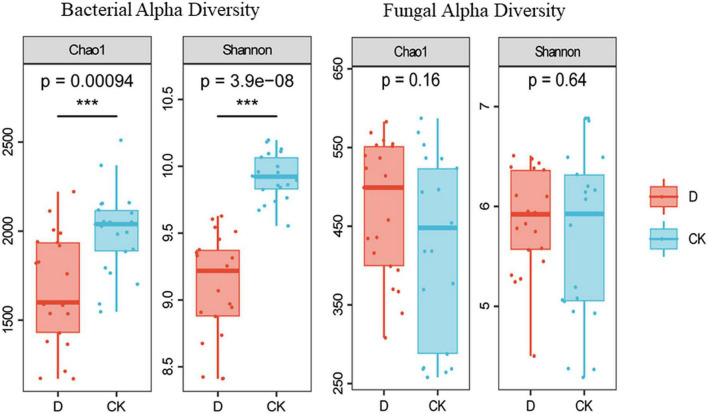
Alpha-diversity of bacterial and fungal communities in the garlic cropping obstacle soil (D) and healthy control soil (CK). *** Indicates statistically significant difference at *P* < 0.001.

The 974,584 16S rRNA gene and 1930,751 ITS sequences were grouped into 67,658 bacterial ASVs and 18,166 fungal ASVs. The richness and diversity indices of the bacteria were lower in the cropping obstacle soil than in the control (*P* < 0.05) (Figure 2).

**FIGURE 3:**
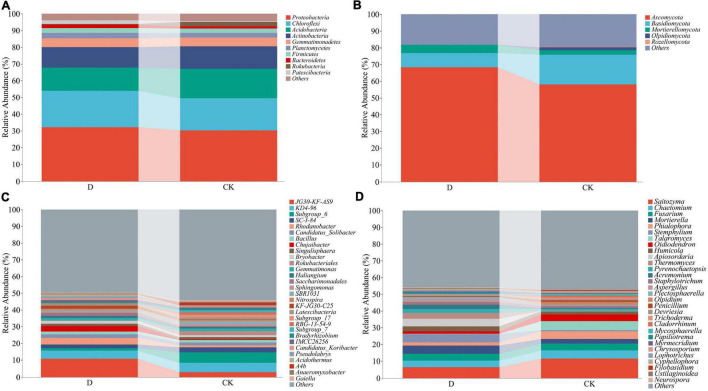
The relative abundances of bacterial and fungal phyla and genera in the garlic cropping obstacle soil (D) and healthy control soil (CK). **(A)** Bacterial phyla, **(B)** fungal phyla, **(C)** bacterial genera, **(D)** fungal genera.

At the phylum level, the bacterial sequences were classified into 36 phyla, out of which the relative abundances of *Proteobacteria*, *Chloroflexi, Acidobacteria*, and *Actinobacteria* were high (Figure 3A and Supplementary Table 2). Out of the 13 fungal phyla, the relative abundances of *Ascomycota* and *Basidiomycota* were high (Figure 3B and Supplementary Table 3). The bacterial sequences were classified into 893 genera and the fungal sequences into 453 genera (Figures 3C,D and Supplementary Tables 4, 5).

**FIGURE 4:**
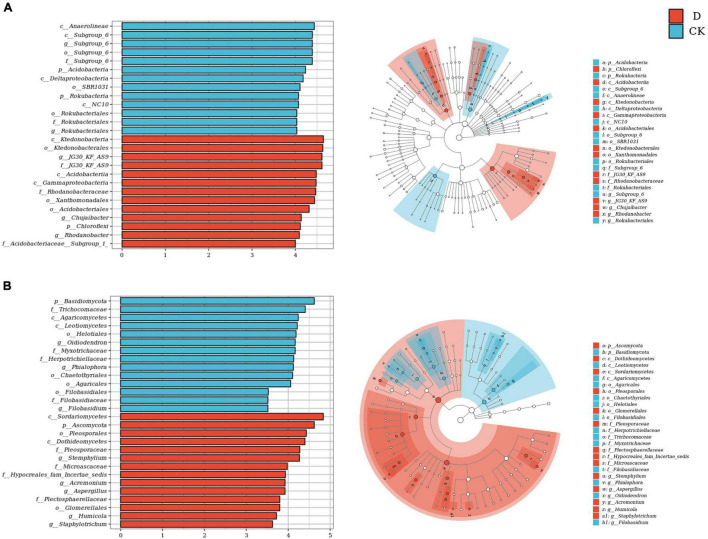
Differential abundances of **(A)** bacterial and **(B)** fungal taxa in the garlic cropping obstacle soil (D) and healthy control soil (CK). The histogram shows taxa with LDA scores ≥ 4.0 for bacterial, and LDA scores ≥ 3.5 for fungal in the linear discriminant (LDA) effect size analysis. The cladogram shows taxonomic differences between D and CK.

Linear discriminant analysis (LDA) effect size (LEfSe) analysis was used to identify differentially abundant taxa. For the bacteria, the taxa with higher relative abundances in the cropping obstacle soil included phylum Chloroflexi, order *Xanthomonadales* and genera *JG30-KF-AS9*, *Chujaibacter* and *Rhodanobacter*; the taxa with lower relative abundances in the cropping obstacle soil included phyla Acidobacteria and Rokubacteria (Figure 4A). For the fungi, the taxa with higher relative abundances in the cropping obstacle soil included genera *Stemphylium* and *Aspergillus*; the taxa with lower relative abundances in the cropping obstacle soil included phylum Basidiomycota and genera *Phialophora* and *Oidiodendron* (Figure 4B).

### The Relationship Between the Microbial Community and Environmental Factors

**FIGURE 5:**
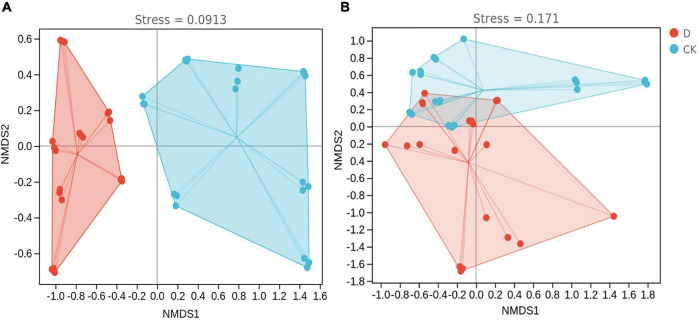
Non-metric multidimensional scaling of **(A)** bacterial and **(B)** fungal communities in the garlic cropping obstacle soil (D) and healthy control soil (CK).

In the non-metric multidimensional scaling (NMDS) based on the Bray-Curtis dissimilarity, the bacterial and fungal communities in the cropping obstacle soil were separated from those in the control soil (Figure 5). In addition, PERMANOVA indicated that the community compositions were different (*P* < 0.05) (Supplementary Table 6).

**FIGURE 6:**
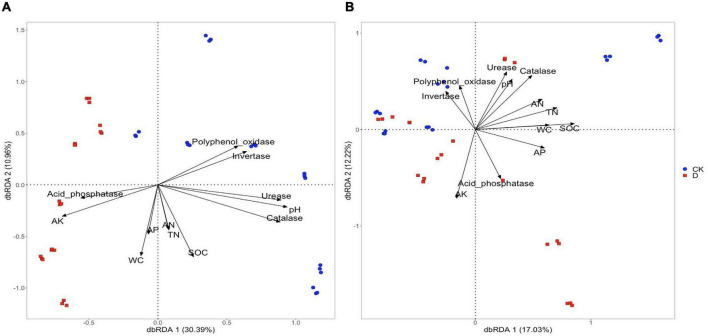
Distance-based redundancy analysis (dbRDA) of environmental factors with **(A)** bacterial and **(B)** fungal taxa in the garlic cropping obstacle soil (D) and healthy control soil (CK).

Based on the distance-based redundancy analysis (dbRDA) analysis, the differences in bacterial community composition across samples were related to soil water content, pH, AP, AK, SOC, TN, polyphenol oxidase and acid phosphatase contents (*P* < 0.001) (Figure 6A and Supplementary Table 7). The differences in community composition between the garlic cropping obstacle soil and the control soil were associated with pH, AK, acid phosphatase, polyphenol oxidase and invertase (Figure 6A and Supplementary Table 7). The differences in fungal community composition across samples were associated with all the measured soil properties, but no clear treatment related associations were detected (Figure 6B and Supplementary Table 7).

### Assembly Processes of Soil Microbial Communities

**FIGURE 7:**
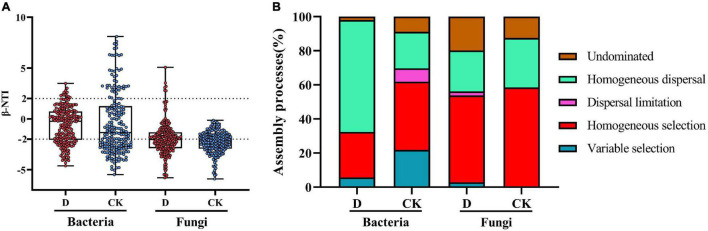
The microbial community assembly processes in the cropping obstacle soil (D) and in the healthy control soil (CK). **(A)** The values of the weighted beta nearest taxon index (βNTI). Horizontal dashed gray lines indicate upper and lower significance thresholds at βNTI = −2 and + 2, respectively. **(B)** The percentages of deterministic processes (homogeneous and variable selection), stochastic processes (dispersal limitation and homogeneous dispersal), and undominated processes.

In the bacterial communities, |β-NTI| > 2 accounted for 32.4% in the cropping obstacle soil and 61.9% in the control (Figure 7A), suggesting that the contribution of deterministic processes to community assembly were lower in the cropping obstacle soil. In the cropping obstacle soil, homogeneous dispersal accounted for 65.6% of the community assembly (Figure 7B). In the control soil, homogeneous selection accounted for 40.0% of the community assembly and variable selection accounted for 21.9%. In the fungal communities, |β-NTI| > 2 accounted for 53.8% in the cropping obstacle soil and 58.6% in the control, suggesting that the assembly processes were primarily deterministic with homogeneous selection as the dominant assembly process (Figure 7B).

## Discussion

Continuous-cropping obstacle soils have received considerable attention in recent years. For example, the biotic and abiotic factors in cropping obstacle of peanut, strawberry and American ginseng have been determined (Li et al., 2014, 2018; Liu et al., 2021). To our knowledge, whether the conclusions based on other crops can be extrapolated to garlic cropping obstacle problem is still not known. We sampled soil and plants in fields with poorly growing garlic plants and noticed that in addition to the evidently lower aboveground biomass, the root biomass and root activity were also lower in the cropping obstacle soil than in the control soil with healthy garlic plants.

Soil properties can directly affect plant health (Wang et al., 2017). In agreement with previous studies (Li et al., 2018; Liu et al., 2021), compared to the control, the garlic cropping obstacle soil was characterized by lower pH and higher available potassium content. Acid stress in pH below 5.5 triggered sensitivity responses in roots, e.g., arrested root growth and death of root tip cells, and acidic soil limited plant growth and the uptake of nutrients from soil (Agegnehu et al., 2016; Gracas et al., 2021). The application of synthetic fertilizers with high level in rice-vegetable rotation has led to a soil acidification (Li et al., 2020; Shen et al., 2021) that may be an important cause for the garlic cropping obstacle. Although differences in the abiotic and biotic characteristic may exist even within close soil environments that share the same geographies (Fierer, 2017), what causes the patchiness of acidification requires further research.

Soil enzyme activities are employed as one of the important indicators of soil quality and fertility (Bastida et al., 2008). In long-period strawberry cropping, the soil enzyme activities decreased and the probability of diseases increased (Li et al., 2018). Similarly, compared to the control, the activities of soil urease, catalase, sucrase and polyphenol oxidase, i.e., enzymes that release soil nutrients for plants, were lower in the garlic cropping obstacle soil. Since soil enzyme activities and pH correlated strongly (Acosta-Martinez and Tabatabai, 2000), the acidification of the garlic cropping obstacle soil could have affected soil enzyme activity. As the soil enzymes mainly originate from soil microorganisms, changes in microbial metabolic activity, lower microbial abundance or changes in the microbial community composition (Zhao et al., 2009; Nannipieri et al., 2012) may have led to the lower enzyme activities.

Continuous cropping obstacles have been associated with lower microbial diversity, a decrease in beneficial microorganisms, and the enrichment of pathogenic microorganisms (Gao et al., 2019, 2021; Tan et al., 2021). According to the insurance hypothesis, biodiversity may act as a buffer against disturbances; in a diverse community, some species are likely to withstand disturbance and carry on functions (Yachi and Loreau, 1999). Alarmingly, in our study, both the richness and the diversity of the bacterial communities were lower in the cropping obstacle soil than in the control. Similar with (Shen et al., 2013), the lower bacterial alpha diversity in cropping obstacle soil may have been due to the lower pH.

Beta diversity analyses showed that the compositions of both the bacterial and fungal communities in the cropping obstacle soil were different from those in the control. Environmental factors affect the microbial communities in soil, with soil pH considered as the master variable in affecting bacterial communities (Fierer and Jackson, 2006). In our study, the differences in the bacterial communities between the cropping obstacle soil and the control were associated with differences in soil pH and AK content. Similarly, pH and AK content were among the main factors associated with bacterial community differences in continuously cropped potato fields (Zhao et al., 2020). Even though pH and nutrient contents affect fungal communities as well (Glassman et al., 2017; Li et al., 2022a), we found no clear associations between fungal community composition and soil properties.

Through studies on the community composition of bacteria and fungi, the differentially distributed taxa were identified by LEfSe analysis. The phylum Chloroflexi and the order Xanthomonadales were enriched in the cropping obstacle soil. Xanthomonadales include plant pathogens of significant economic and agricultural impact (Bayer-Santos et al., 2019). The higher relative abundance of Chloroflexi was mostly due to the uncultured genus JG30-KF-AS9 that can adapt to acidic soil and be detrimental to enzyme activities (Wang et al., 2019). Thus, the enrichment of JG30-KF-AS9 may be connected with the lower enzyme activities in the garlic cropping obstacle soil. *Chujaibacter* and *Rhodanobacter* which were actively developing in the presence of mineral fertilizers were acidophilic microorganisms participating in the nitrogen cycle (Semenov et al., 2020), it is speculated that the increase of these microorganisms may be related to soil acidification. Likewise, the fungal genera *Stemphylium* and *Aspergillus* that were enriched in the cropping obstacle soil include plant pathogens (Syed et al., 2020; Dumin et al., 2021). For example, *Stemphylium* spp. caused garlic leaf spot disease (Dumin et al., 2021). Genus *Oidiodendron*, one of the most widely investigated ericoid mycorrhizal fungi that had plant growth promoting characteristics (Wei et al., 2016; Baba et al., 2021), was depleted in the cropping obstacle soil. Thus, the enrichment of Xanthomonadales, *Stemphylium* and *Aspergillus* and depletion of *Oidiodendron* may have contributed to the poor growth of garlic in the cropping obstacle soil.

Uncovering the microbial community assembly processes is a challenging task (Stegen et al., 2013; Luan et al., 2020). Our results showed that the bacteria assembly processes in garlic cropping obstacle soil was governed by homogeneous dispersal, a stochastic process that homogenizes the bacterial community structure and causes low compositional turnover (Stegen et al., 2013). In the control, the community assembly was characterized by deterministic processes. Root exudates increase available resources that may facilitate the recruitment and selection of bacterial taxa (Li et al., 2022b), which may explain why deterministic processes governed assembly processes in the control soil with vigorously growing garlic plants.

Homogeneous selection was regarded as a factor leading to a stable state after disturbance in progressive succession of communities (Dini-Andreote et al., 2015). Together with the diversity and environmental factor association results, the dominance of homogeneous selection in both the cropping obstacle and control soils suggested that the fungal communities were more resilient than the bacterial communities.

## Summary

Cropping obstacle soil was characterized by acidification and lower enzyme activities except for the activity of acid phosphatase. The lower bacterial richness and diversity, and the enrichment of plant pathogens may have contributed to the garlic cropping obstacle. The lower enzyme activity and microbial community differences were associated with lower pH in the cropping obstacle soil. The correlations among plant growth, soil properties and microbial communities cannot reveal cause and effect, thus discovering the exact mechanisms behind the cropping obstacle phenomenon require further controlled experiments.

## Data Availability Statement

The data presented in the study are deposited in the National Center for Biotechnology Information (NCBI) with the accession number SRP348212.

## Author Contributions

JY, QC, and ZS conceived and designed this experiment. JY, ZW, YL, and DC collected samples and carried out experiments. YG, KZ, XY, HL, and PP performed the bioinformatics and statistical analysis. JY, XZ, QC, and PP wrote and revised the manuscript. All authors read and approved the final manuscript.

## Conflict of Interest

HL was employed by the CNPC Chuanqing Drilling Engineering Co., Ltd. The remaining authors declare that the research was conducted in the absence of any commercial or financial relationships that could be construed as a potential conflict of interest.

## Publisher’s Note

All claims expressed in this article are solely those of the authors and do not necessarily represent those of their affiliated organizations, or those of the publisher, the editors and the reviewers. Any product that may be evaluated in this article, or claim that may be made by its manufacturer, is not guaranteed or endorsed by the publisher.

## Footnotes

1
https://docs.qiime2.org/2019.4/tutorials/

2
http://www.r-project.org
