# Supplementary material for: The Cropping Obstacle of Garlic Was Associated With Changes in Soil Physicochemical Properties, Enzymatic Activities and Bacterial and Fungal Communities
Source: Front Microbiol. 2022 Mar 30;13:828196. doi: 10.3389/fmicb.2022.828196 (PMC9006102; doi:10.3389/fmicb.2022.828196)
Supplement: Supplementary file 1 [file Table_1.DOCX]

**Supplementary materials list**

**Supplementary tables**

**Supplementary Table S1** Correlations between the properties of garlic plants and soil.

**Supplementary Table S2** The relative abundances of bacterial phyla in the garlic cropping obstacle soil (D) and healthy control soil (CK).

**Supplementary Table S3** The relative abundances of fungal phyla in the garlic cropping obstacle soil (D) and healthy control soil (CK).

**Supplementary Table S4** The relative abundances of thirty most abundant bacterial genera in the garlic cropping obstacle soil (D) and healthy control soil (CK).

**Supplementary Table S5** The relative abundances of thirty most abundant fungal genera in the garlic cropping obstacle soil (D) and healthy control soil (CK).

**Supplementary Table S6** PERMANOVA analysis of bacterial and fungal community compositions based on Bray-Curtis dissimilarities.

**Supplementary Table S7** Correlations between bacterial and fungal community compositions and soil properties.

**Table S1** Correlations between the properties of garlic plants and soil.

|  | Shoot length | Maximum root length | Shoot fresh weight | Root fresh weight | Root activity |
| --- | --- | --- | --- | --- | --- |
| pH | 0.777** | 0.737** | 0.752** | 0.745** | 0.705** |
| WC | -0.199 | -0.222 | -0.150 | -0.166 | -0.142 |
| AP | -0.173 | -0.150 | -0.184 | -0.258 | -0.175 |
| AK | -0.702** | -0.694** | -0.685** | -0.570** | -0.761** |
| SOC | 0.244 | 0.200 | 0.219 | 0.136 | 0.225 |
| TN | 0.031 | -0.019 | 0.059 | -0.056 | 0.023 |
| AN | -0.008 | -0.054 | 0.067 | 0.015 | -0.063 |

** Statistically significant correlation at p<0.01 (two-sided). WC, soil water content; AP, available phosphorus content; AK, available potassium content; AN, available nitrogen content; TN, total nitrogen content; SOC, soil organic carbon content.

**Table S2** The relative abundances of bacterial phyla in the garlic cropping obstacle soil (D) and healthy control soil (CK).

| Taxonomy | Relative Abundance | |
| --- | --- | --- |
|  | D | CK |
| Proteobacteria | 0.32±0.06% | 0.30±0.03% |
| Chloroflexi | 0.22±0.04% | 0.19±0.04% |
| Acidobacteria | 0.14±0.03% | 0.17±0.02% |
| Actinobacteria | 0.12±0.02% | 0.13±0.03% |
| Gemmatimonadetes | 0.05±0.01% | 0.06±0.02% |
| Planctomycetes | 0.03±0.02% | 0.03±0.02% |
| Firmicutes | 0.03±0.01% | 0.03±0.01% |
| Bacteroidetes | 0.02±0.01% | 0.01±0% |
| Rokubacteria | – | 0.03±0.01% |
| Patescibacteria | 0.02±0.02% | 0.01±0.01% |
| Others | 0.04±0.02% | 0.04±0.01% |

The results are average ± standard deviation. – indicates relative abundance < 0.01%.

**Table S3** The relative abundances of fungal phyla in the garlic cropping obstacle soil (D) and healthy control soil (CK).

| Taxonomy | Relative Abundance | |
| --- | --- | --- |
|  | D | CK |
| Ascomycota | 0.68±0.09% | 0.58±0.13% |
| Basidiomycota | 0.09±0.07% | 0.18±0.12% |
| Mortierellomycota | 0.05±0.05% | 0.03±0.04% |
| Olpidiomycota | – | 0.02±0.04% |
| Others | 0.18±0.08% | 0.19±0.07% |

The results are average ± standard deviation. – indicates relative abundance < 0.01%.

**Table S4** The relative abundances of thirty most abundant bacterial genera in the garlic cropping obstacle soil (D) and healthy control soil (CK).

| Taxonomy | Relative Abundance | | |
| --- | --- | --- | --- |
|  | D | CK |  |
| JG30-KF-AS9 | 0.11±0.03% | 0.03±0.03% |  |
| KD4-96 | 0.05±0.02% | 0.06±0.01% |  |
| Subgroup_6 | 0.01±0.01% | 0.06±0.02% |  |
| SC-I-84 | 0.02±0.01% | 0.03±0.01% |  |
| Rhodanobacter | 0.04±0.01% | 0.01±0.02% |  |
| Candidatus_Solibacter | 0.02±0.01% | 0.02±0.01% |  |
| Bacillus | 0.02±0.01% | 0.02±0.00% |  |
| Chujaibacter | 0.03±0.04% | – |  |
| Singulisphaera | 0.02±0.01% | 0.01±0.01% |  |
| Bryobacter | 0.02±0.01% | 0.01±0.00% |  |
| Rokubacteriales | – | 0.03±0.01% |  |
| Gemmatimonas | 0.01±0.00% | 0.01±0.01% |  |
| Haliangium | 0.01±0.00% | 0.01±0.00% |  |
| Saccharimonadales | 0.02±0.01% | – |  |
| Sphingomonas | 0.01±0.00% | 0.01±0.00% |  |
| SBR1031 | – | 0.02±0.01% |  |
| Nitrospira | 0.01±0.00% | 0.01±0.00% |  |
| KF-JG30-C25 | 0.02±0.02% | – |  |
| Latescibacteria | 0.01±0.01% | 0.01±0.00% |  |
| Subgroup_17 | – | 0.02±0.01% |  |
| RBG-13-54-9 | 0.00±0.01% | 0.01±0.01% |  |
| Subgroup_7 | – | 0.01±0.01% |  |
| Bradyrhizobium | 0.01±0.00% | 0.01±0.00% |  |
| IMCC26256 | 0.01±0.00% | 0.01±0.00% |  |
| Candidatus_Koribacter | 0.01±0.01% | – |  |
| Pseudolabrys | 0.01±0.00% | – |  |
| Acidothermus | 0.01±0.01% | – |  |
| A4b | – | 0.01±0.01% |  |
| Anaeromyxobacter | 0.01±0.00% | 0.01±0.00% |  |
| Gaiella | – | 0.01±0.00% |  |
| Others | 0.5±0.03% | 0.54±0.03% |  |

The results are average ± standard deviation. – indicates the relative abundance < 0.01%.

**Table S5** The relative abundances of thirty most abundant fungal genera in the garlic cropping obstacle soil (D) and healthy control soil (CK).

| Taxonomy | Relative Abundance | | |
| --- | --- | --- | --- |
|  | D | CK |  |
| Saitozyma | 0.06±0.06% | 0.11±0.12% |  |
| Chaetomium | 0.04±0.03% | 0.05±0.06% |  |
| Fusarium | 0.04±0.04% | 0.04±0.03% |  |
| Mortierella | 0.05±0.05% | 0.03±0.04% |  |
| Phialophora | 0.02±0.04% | 0.05±0.06% |  |
| Stemphylium | 0.05±0.08% | 0.01±0.01% |  |
| Talaromyces | – | 0.05±0.11% |  |
| Oidiodendron | 0.01±0.03% | 0.04±0.08% |  |
| Humicola | 0.03±0.02% | 0.02±0.02% |  |
| Apiosordaria | 0.05±0.11% | – |  |
| Thermomyces | 0.04±0.08% | – |  |
| Pyrenochaetopsis | 0.02±0.02% | 0.01±0.01% |  |
| Acremonium | 0.02±0.02% | 0.01±0.00% |  |
| Staphylotrichum | 0.02±0.02% | 0.01±0.02% |  |
| Aspergillus | 0.02±0.03% | – |  |
| Plectosphaerella | 0.01±0.01% | 0.01±0.01% |  |
| Olpidium | – | 0.02±0.04% |  |
| Penicillium | 0.01±0.01% | 0.01±0.01% |  |
| Devriesia | 0.01±0.01% | 0.01±0.01% |  |
| Trichoderma | – | 0.01±0.01% |  |
| Cladorrhinum | – | 0.01±0.03% |  |
| Mycosphaerella | 0.00±0.01% | 0.01±0.02% |  |
| Papiliotrema | 0.01±0.03% | 0.00±0.01% |  |
| Myrmecridium | 0.01±0.01% | – |  |
| Chrysosporium | – | 0.01±0.01% |  |
| Lophotrichus | 0.01±0.02% | – |  |
| Cyphellophora | 0.01±0.01% | – |  |
| Filobasidium | – | 0.01±0.02% |  |
| Ustilaginoidea | 0.00±0.01% | – |  |
| Neurospora | 0.01±0.02% | – |  |
| Others | 0.46±0.15% | 0.47±0.13% |  |

The results are average ± standard deviation. – indicates relative abundance < 0.01%.

**Table S6.** PERMANOVA analysis of bacterial and fungal community compositions based on Bray-Curtis dissimilarities.

|  | R^2^ | F. Model | Pr (>F) | Permutations |
| --- | --- | --- | --- | --- |
| Bacteria | 0.19 | 9.75 | 0.001 | 999 |
| Fungi | 0.07 | 3.17 | 0.001 | 999 |

**Table S7**. Correlations between bacterial and fungal community compositions and soil properties.

|  | Bacteria | | | Fungi | | |
| --- | --- | --- | --- | --- | --- | --- |
|  | F | Pr(>F) |  | F | Pr(>F) |  |
| WC | 9.428 | 0.001 | *** | 10.2794 | 0.001 | *** |
| pH | 31.5509 | 0.001 | *** | 9.1899 | 0.001 | *** |
| AP | 5.0133 | 0.001 | *** | 7.7326 | 0.001 | *** |
| AK | 10.0723 | 0.001 | *** | 8.5922 | 0.001 | *** |
| SOC | 5.6119 | 0.001 | *** | 6.8737 | 0.001 | *** |
| TN | 6.692 | 0.001 | *** | 6.9892 | 0.001 | *** |
| AN | 1.9986 | 0.045 | * | 3.2079 | 0.001 | *** |
| Catalase | 3.4806 | 0.002 | ** | 4.077 | 0.001 | *** |
| Urease | 1.4129 | 0.168 |  | 2.7932 | 0.001 | *** |
| Polyphenol_oxidase | 4.8321 | 0.001 | *** | 6.2264 | 0.001 | *** |
| Acid_phosphatase | 4.2146 | 0.001 | *** | 6.6257 | 0.001 | *** |
| Invertase | 1.8735 | 0.065 |  | 2.3139 | 0.008 | ** |

WC, soil water content; AP, available phosphorus content; AK, available potassium content; AN, available nitrogen content; TN, total nitrogen content; SOC, soil organic carbon content.

* *P*<0.05, ** *P*<0.01, *** *P*<0.001.
